# Impacts of Population Size and Domestication Process on Genetic Diversity and Genetic Load in Genus *Ovis*

**DOI:** 10.3390/genes14101977

**Published:** 2023-10-23

**Authors:** Dongfeng Wang, Hosein Salehian-Dehkordi, Langda Suo, Fenghua Lv

**Affiliations:** 1 CAS Key Laboratory of Animal Ecology and Conservation Biology, Institute of Zoology, Chinese Academy of Sciences (CAS), Beijing 100101, China; atongsa@163.com; 2 College of Life Sciences, University of Chinese Academy of Sciences (UCAS), Beijing 100049, China; 3 College of Animal Science and Technology, China Agricultural University, Beijing 100193, China; hosein_salehi6@yahoo.com; 4 Institute of Animal Science, Tibet Academy of Agricultural and Animal Husbandry Sciences, Lhasa 850009, China; sonaada10@163.com

**Keywords:** *Ovis*, genetic diversity, genetic load, effective population, domestication, improvement

## Abstract

In theoretical biology, a prevailing hypothesis posits a profound interconnection between effective population size (*N*_e_), genetic diversity, inbreeding, and genetic load. The domestication and improvement processes are believed to be pivotal in diminishing genetic diversity while elevating levels of inbreeding and increasing genetic load. In this study, we performed a whole genome analysis to quantity genetic diversity, inbreeding, and genetic load across seven wild *Ovis* species and five domesticated sheep breeds. Our research demonstrates that the genetic load and diversity of species in the genus *Ovis* have no discernible impact on recent *N*_e_, and three species within the subgenus *Pachyceros* tend to carry a higher genetic load and lower genetic diversity patterns. The results coincide with these species’ dramatic decline in population sizes within the subgenus *Pachyceros* ~80–250 thousand years ago. European mouflon presented with the lowest *N*_e_, lower genetic diversity, and higher individual inbreeding coefficient but a lower genetic load (missense and LoF). This suggests that the small *N*_e_ of European mouflon could reduce harmful mutations compared to other species within the genus *Ovis*. We showed lower genetic diversity in domesticated sheep than in Asiatic mouflon, but counterintuitive patterns of genetic load, i.e., lower weak genetic load (missense mutation) and no significant difference in strong genetic load (LoF mutation) between domestic sheep and Asiatic mouflon. These findings reveal that the “cost of domestication” during domestication and improvement processes reduced genetic diversity and purified weak genetic load more efficiently than wild species.

## 1. Introduction

The effective population size (*N*_e_) describes the population size caused by genetic drift under the random sampling of genetic variants in a finite population, which plays a pivotal role in molding the dynamics of genetic drift and natural selection within populations [1,2]. Previous studies have shown smaller effective population size (*N*_e_) values correlated with reductions in genetic diversity and increases in mutation burden (genetic variations are more likely to be deleterious than beneficial and a major cause of variation in fitness-related traits among individuals), respectively [1,2]. Nevertheless, the relationships between effective population size (*N*_e_), genetic diversity, and genetic load were changeable, as demonstrated in prior studies among certain species [3,4,5]. 

Compared with wild species, the domestication process and artificial selection were imposed for domesticated species. One hypothesized cost of domestication suggested that the processes of domestication and improvement result in an increased mutation burden [6]. Recent studies supported the hypothesis that domesticated species had higher genetic load than their wild ancestors [7,8,9,10]. However, domestication and improvement processes may affect patterns of the mutation burden across various species. The domestication process led to the purging of large-effect deleterious mutations while amplifying the burden of small-effect mutations in maize [10]. The improvement processes led to a reduction of genetic diversity and increased the genetic load in chickens [7], dogs [8], and crops [9]. It is essential to note that the relationship between domestication, improvement, and genetic load is multifaceted and influenced by many factors [6]. Therefore, clarifying the impacts of population size, the domestication process, and the improvement in genetic diversity and load brought a considerable challenge.

Wild and domesticated sheep in the genus *Ovis* provided an excellent model by which to investigate relationships between effective population size, the process of domestication and improvement, and genetic load/genetic diversity. Seven wild species in the genus *Ovis* exhibit a wide intercontinental distribution in Eurasia and North America [11], and the main driving force of evolution is nature selection, except for in the case of European mouflon (*Ovis musimon*, EMUF) [12,13]. The domesticated sheep originated from Asiatic mouflon (*Ovis orientalis*, AMUF) approximately 11,000 years ago in the Fertile Crescent [14,15,16], and the process of improvement after domestication reshaped the genomic features of sheep [17] and produced various phenotypes [18]. In this study, we focused on exploring the effects of effective population size (*N*_e_) on the genetic diversity and genetic load of wild *Ovis* species and further investigate the impacts of the domestication and improvement process on the genetic diversity and genetic load on AMUF, three native breeds (Tibetan sheep, TIB; Finnsheep, FIN; Shetland sheep, SHE) and two improved breeds (super-fine-wool Merino, MSF; fine-wool Merino, MFW), of which Tibetan sheep is a native breed which has adapted to the Qinghai–Tibetan Plateau environments, and Finnsheep and Shetland sheep are primitive breeds on the periphery of northwest Europe [14].

## 2. Materials and Methods

### 2.1. Data Collecting

Whole genome sequencing data of 123 individuals were retrieved from NCBI and a previous study [19]. The dataset encompassed three categories: (i) 71 wild sheep (3 *Ovis musimon*, 31 *Ovis orientalis*, 9 *Ovis vignei*, 8 *Ovis ammon*, 8 *Ovis nivicola*, 6 *Ovis dalli,* and 6 *Ovis canadensis*); (ii) 32 native individuals (15 Tibetan sheep, 10 Finnland sheep, and 7 Shetland sheep); and (iii) 20 improved samples (20 Chinese Merino) (Table 1, Supplementary Table S1). 

### 2.2. SNP Calling

Whole genome sequencing (WGS) data were initially trimmed using Trimmomatic v0.3917 [20] and subsequently aligned to the sheep reference genome Oar_rambouillet_v1.0 (GCF_002742125.1) (https://www.ncbi.nlm.nih.gov/datasets/genome/GCF_002742125.1/, last accessed on 17 June 2023) via BWA v0.7.17-r1188 mem [21] with default parameters. We further filtered BAM files with the *MarkDuplicates* module of Picard v2.18.12 (http://broadinstitute.github.io/picard/, last accessed on 17 June 2023) to remove duplicates and detected single nucleotide polymorphisms (SNPs) with the GATK best-practice recommendations [22]. We first generated the GVCF file of each sample via the *HaplotypeCaller* module. Secondly, all GVCF files were merged via the *CombineGVCFs* module, and the raw SNPs were called using the *GenotypeGVCFs* module. The biallelic SNPs were selected by the *SelectVariants* module in GATK and further by the *VariantFiltering* module of the GATK with the following parameters: “QUAL < 30.0||QD < 2.0||MQ < 40.0||FS > 60.0||SOR > 3.0||MQRankSum < −12.5||ReadPosRankSum < −8.0”. We further trimmed the SNPs using vcftools v0.1.17 [23] with the following criteria: (i) missing rate > 0.90; (ii) minor allele frequency (MAF) > 0.01; (iii) SNPs in autosomes. A total of 60,369,351 autosomal SNPs were identified.

### 2.3. Estimating Index of Genetic Diversity and Calculating Runs of Homozygosity (ROH)

Nucleotide diversity (π) was calculated using all sites with VCFtools v0.1.17 [23] for seven wild species and five domestic sheep breeds, with a sliding window of 2 Mb and a 1 Mb sliding step. We further calculated the heterozygosity of individuals using PLINK v1.90b6.26 [24] with the “-het” option.

The ROH were identified using detectRUNS v0.9.6 [25] with the following parameters: (i) maxOppRun = 0 [26]; (ii) maxMissRun = 0 [26]; (iii) minSNP = 230 [27]; (iv) –homozyg-kb 250 [26]; and (v) maxGap = 10^6^ [26]. Then, the *F*_ROH_ was calculated as the percentage of the autosome genome covered by ROH for each sample [28].

$$F_{ROH}=\frac{\sum_{i}L_{{ROH}_{i}}}{L_{autosome}},$$

where *L_ROHi_* is the length of *ROH_i_* of individual *i*; and *L*_autosome_ is the autosomal genome length.

### 2.4. Estimates of Effective Population Size (N_e_)

We estimated the recent effective population size (*N*_e_) of all seven wild sheep species by SNeP v1.1 [29], which inferred the *N*_e_ based on the equation $E\left(r|2\right)=1/\left(1+4N_{e}c\right)$, where *c* is recombination rate and *r*^2^ is the correlation coefficient between pairs of loci [30]. We implemented the analysis with the default setting except for sample size and recombination rates. The sample size was corrected to 2, and the recombination rate was adjusted using the “svedf” option. We estimated the *N*_e_ 100 generations ago to scale population dynamics using the different SNP marker distance bins. 

### 2.5. Estimate of Genetic Load

To estimate mutation burden, we first inferred the ancestral allelic state of each SNP following the EPO pipeline with modification (https://ftp.ensembl.org/pub/release-65/fasta/ancestral_alleles/pan_troglodytes_ancestor_CHIMP2.1.4_e65.README, last accessed on 12 June 2023). First, the goat reference genomic sequences (GCA_001704415.2) (https://www.ncbi.nlm.nih.gov/datasets/genome/GCF_001704415.2/, last accessed on 12 June 2023) were aligned against the sheep genome (GCF_002742125.1) (https://www.ncbi.nlm.nih.gov/datasets/genome/GCF_002742125.1/, last accessed on 17 June 2023) using minimap2 v2.24-r1122 [31]. Second, goat SNP was called via bcftools v1.16 [32] with default setting and merged with the dataset generated from all the domestic and wild sheep individuals. Finally, we filtered out the sites with different alleles among goats and *Ovis* and defined each SNP’s ancestral state as the goats’ allele state. 

Subsequently, we inferred the deleterious mutations of the polarized SNPs dataset using SnpEff v5.1d [33]. The analyses were run using the sheep reference genome (GCF_002742125.1) (https://www.ncbi.nlm.nih.gov/datasets/genome/GCF_002742125.1/, last accessed on 17 June 2023) annotation file (https://ftp.ncbi.nlm.nih.gov/genomes/all/annotation_releases/9940/103/GCF_002742125.1_Oar_rambouillet_v1.0/GCF_002742125.1_Oar_rambouillet_v1.0_genomic.gff.gz, last accessed on 17 June 2023) with the “-lof” option. Deleterious mutation SNPs were categorized as loss of function (“LOF”) and missense mutations. We first counted the total heterozygotes and derived homozygotes per individual for LoF and missense sites to investigate how mutation load varies across species and populations. We assessed the whole genetic load by calculating the number of derived alleles [34]. The potential and realized genetic loads [35] were estimated based on the number of heterozygous and homozygous mutations. 

## 3. Results

### 3.1. Genetic Diversity Pattern

We collected whole genome resequencing data for 123 individuals from seven wild species and five domesticated sheep breeds (Supplementary Table S1). A total of 60,369,351 high-quality SNPs were identified. 

**Figure 1:**
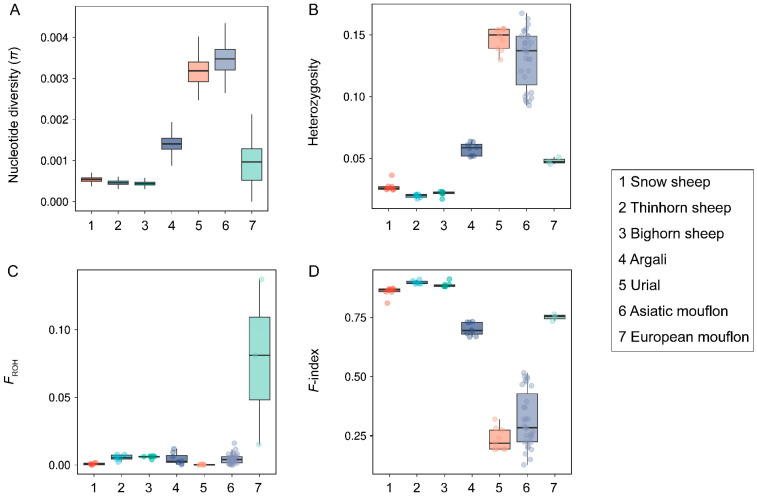
The genetic diversity and individual coefficients of seven wild *Ovis* species: (**A**) nucleotide diversity (π) of seven wild species within the genus *Ovis*; (**B**) heterozygosity of seven wild species within the genus *Ovis*; (**C**) individuals inbreeding coefficients (*F*_ROH_) of seven wild species within the genus *Ovis*; (**D**) *F*-index of seven wild species within genus *Ovis*.

With genus *Ovis*, we observed extensive variation of genetic diversity among species (Figure 1), and Asiatic mouflon and Bighorn sheep showed the highest π (0.00326) and the lowest π (0.00043) (Figure 1A), respectively. Four species (European mouflon, Asiatic mouflon, Urial, and Argali) exhibited higher genetic diversity and lower inbreeding compared to three species of subgenus *Pachyceros* (Snow sheep, Thinhorn sheep, and Bighorn sheep) (Figure 1). More similar patterns of π (0.00043–0.00053), heterozygosity (0.0196–0.0270), *F*_ROH_ (0.0008–0.0059), and *F*-index (0.8590–0.8980) were observed within three species in subgenus *Pachyceros* (Figure 1 and Supplementary Table S3), which suggested that three species in subgenus *Pachyceros* may have similar demographic history. Within the subgenus *Pachyceros*, Snow sheep (SOWS) showed the lowest *F*_ROH_ (0.0008), which was significantly lower than that found for the other two American wild species (both *p* = 0.002, Wilcoxon) (Figure 1C). The genetic diversity (π and heterozygosity) and inbreeding coefficients (*F*_ROH_ and *F*-index) of Argali are at an intermediate level between Moufloniforms and Pachyceriforms (Figure 1). However, an exception was observed in Moufloniforms; the European mouflon possessed the lowest genetic diversity and highest inbreeding among four species (European mouflon, Asiatic mouflon, Urial, and Argali) (Figure 1A,B,D) and exhibited the highest *F*_ROH_ (0.0778) among all the wild sheep (Figure 1C). In contrast, Urial and Asiatic mouflon within Moufloniforms displayed the lowest *F*_ROH_ (0.0003) and *F*-index (0.2344) and the highest π (0.00326) and heterozygosity (0.1467) (Figure 1). The results indicate a specific evolutionary history of European mouflon. 

### 3.2. Genetic Load Comparisons among Wild Species within the Genus Ovis

**Figure 2:**
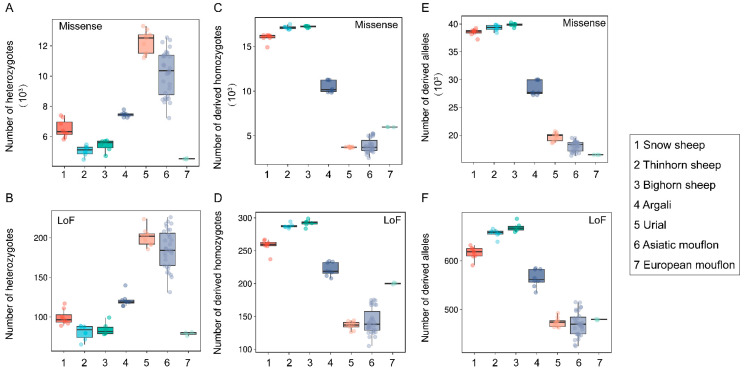
The genetic load of seven wild species within the genus *Ovis*: (**A**) the number of heterozygotes-derived missense variants; (**B**) the number of heterozygotes-derived LoF variants; (**C**) the number of homozygous-derived missense variants; (**D**) the number of homozygous-derived LoF variants; (**E**) total number of allelic-derived missense variants; (**F**) total number of allelic-derived LoF variants.

Extensive variation in both heterozygotes- and homozygous-derived missense or LoF variants was observed among the seven species (Figure 2). Three species of subgenus *Pachyceros* showed similar patterns of genetic load and possessed lower heterozygosity-derived missense and LoF variants than Argali, Urial, and Asiatic mouflon (Figure 2A,B). In contrast, higher homozygous-derived missense and LoF variant genotypes and allelic-derived variants were observed in three species of subgenus *Pachyceros* than Argali, Urial, and Asiatic mouflon (Figure 2C–F). We also observed a specific pattern in European mouflon contrasting with the other species within Moufloniforms, which possessed the lowest heterozygous-derived variants (Figure 2A,B). We further characterized the potential load and the realized load using the number of heterozygotes- and homozygotes-derived variants with the methodologies described in Bertorelle et al. [36] and Grossen et al. [37]. Our results unveiled an opposite genetic load pattern between subgenus *Pachyceros* and other species within *Ovis*; Snow sheep, Thinhorn sheep, and Bighorn sheep showed lower potential loads and higher realized loads than Argali, Urial, and Asiatic mouflon (Figure 2A–D). Considering the overall mutation load (Figure 2E,F), our results indicated that subgenus *Pachyceros* has a higher mutation load. 

### 3.3. Genetic Load and Effective Population Size (N_e_)

**Figure 3:**
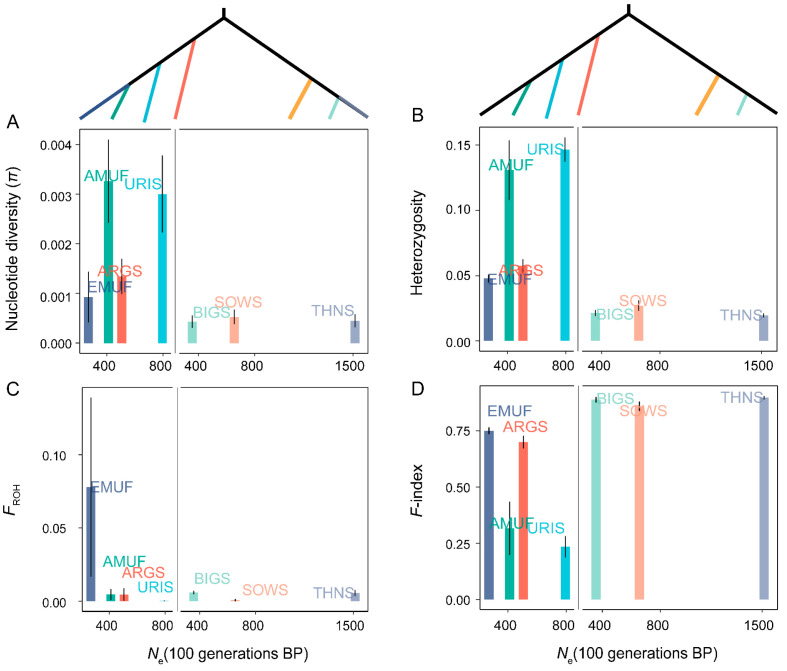
The relationship between *N*_e_ and genetic diversity among seven wild species within the genus *Ovis*: (**A**) nucleotide diversity (π); (**B**) heterozygosity; (**C**) individuals inbreeding coefficients (*F*_ROH_); (**D**) *F*-index.

We estimated the *N*_e_ of seven species over the last 100 generations using the SNeP method to assess the impact of recent effective population size. Genetic diversity increases with effective population size under the neutral theory [3]. We hypothesized that lower *N*_e_ would coincide with reduced genetic diversity and increased genetic load in wild sheep. However, our analysis did not find a correlation between *N*_e_ and genetic diversity across all seven *Ovis* species (Figure 3). Interestingly, we observed different *N*_e_ of three species in subgenus *Pachyceros* (*N*_e-BIG_ = 356.73; *N*_e-SOWS_ = 654.79; *N*_e-THNS_ = 1513.19) and similar genetic diversity and individual inbreeding coefficient patterns (Figure 1 and Figure 3).

**Figure 4:**
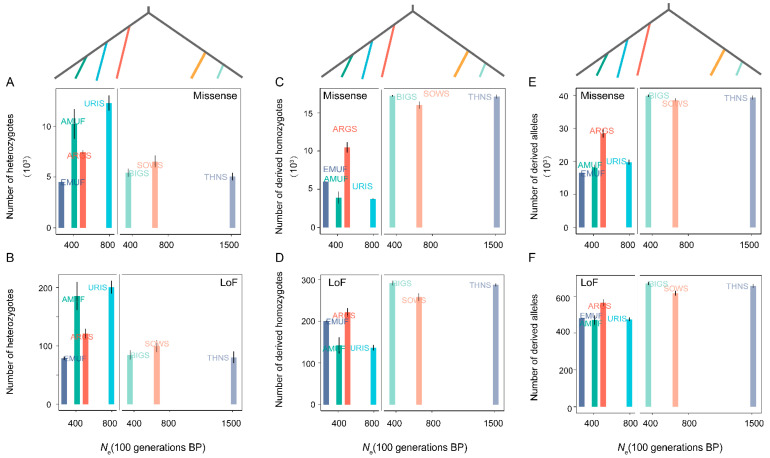
The relationship between genetic load and *N*_e_ among seven wild species within the genus *Ovis***:** (**A**) the number of heterozygotes-derived missense variants; (**B**) the number of heterozygotes-derived LoF variants; (**C**) the number of homozygous-derived missense variants; (**D**) the number of homozygous-derived LoF variants; (**E**) total number of allelic-derived missense variants; (**F**) total number of allelic-derived LoF variants.

A comparatively similar pattern was detected in the genetic load across seven species over the last 100 generations. We did not observe any significant correlation between *N*_e_ and the number of heterozygotes, derived homozygotes, and the total number of derived allelic variants for both Missense and LoF mutations (Figure 4). To eliminate the potential effect of phylogeny, we further investigate the relationships between *N*_e_ and genetic load within Pachyceriforms and Moufloniforms/Argaliforms, respectively. We also found no correlation between *N*_e_ and the number of heterozygotes-derived missense variants, the number of heterozygotes-derived LoF variants, the number of homozygous-derived LoF variants, the total number of allelic-derived missense variants, and the total number of allelic-derived LoF variants (Figure 4). Similar to the genetic diversity pattern, three species in the subgenus *Pachyceros* (*N*_e-BIG_ = 356.73; *N*_e-SOWS_ = 654.79; *N*_e-THNS_ = 1513.19) showed similar values of genetic load. 

### 3.4. Relationships of Domestication and Improvement Process with Genetic Diversity and Genetic Load

To investigate the effects of the domestication and improvement process on genetic diversity and genetic load, we further calculated the genetic diversity and genetic load of native breeds (Finnsheep, Shetland sheep, and Tibetan sheep) and improved breeds (Chinese Merino sheep). 

**Figure 5:**
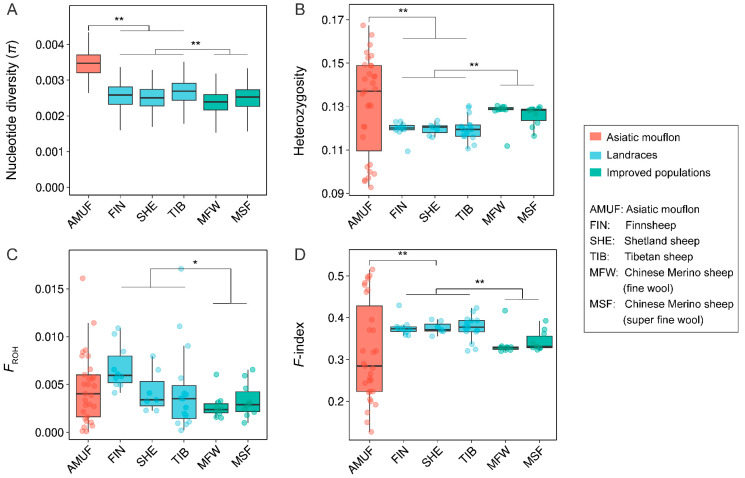
The genetic diversity and individual coefficients of Asiatic mouflon, native, and improved sheep breeds: (**A**) nucleotide diversity (π) of Asiatic mouflon, native, and improved sheep breeds; (**B**) heterozygosity of Asiatic mouflon, native, and improved sheep breeds; (**C**) individuals inbreeding coefficients (*F*_ROH_) of Asiatic mouflon, native, and improved sheep breeds; (**D**) *F*-index of Asiatic mouflon, native, and improved sheep breeds, *: *p* < 0.05; **: *p* < 0.01.

**Figure 6:**
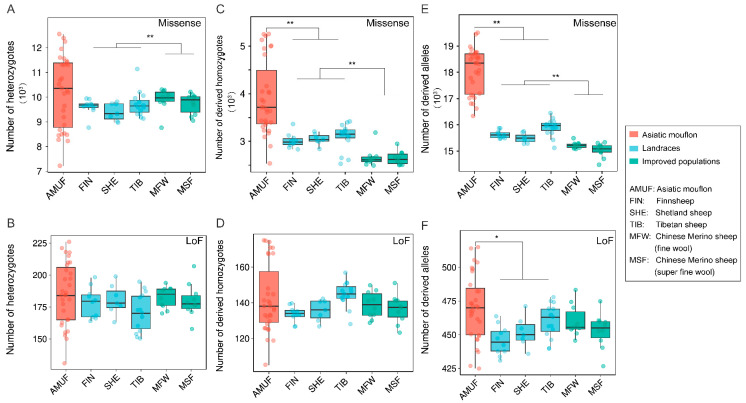
The genetic load of Asiatic mouflon, native, and improved sheep breeds: (**A**) the number of heterozygotes-derived missense variants; (**B**) the number of heterozygotes-derived LoF variants; (**C**) the number of homozygous-derived missense variants; (**D**) the number of homozygous-derived LoF variants; (**E**) total number of allelic-derived missense variants; (**F**) total number of allelic-derived LoF variants, *: *p* < 0.05; **: *p* < 0.01.

We found that the Asiatic mouflon population presented significantly higher nucleotide diversity (π) than both native sheep (*p* < 0.001) and improved sheep (*p* < 0.001), and the native sheep possessed higher π than the improved sheep (*p* < 0.001) (Figure 5A). Additionally, the Asiatic mouflon population showed higher heterozygosity than the native populations (*p* < 0.01), which showed lower heterozygosity than the improved sheep breeds (*p* < 0.001). However, the heterozygosity of the improved species was significantly higher than that of the native breeds (*p* < 0.001) and not considerably different from Asiatic mouflon (*p* > 0.05) (Figure 5B). *F*_ROH_ and F-index were lower in improved breeds than in native breeds (*p* < 0.05) (Figure 5C,D), and the result conflicts with the hypothesis of improved processes decreasing genetic diversity. We also evaluated the impact of domestication and improvement processes on genetic load for missense and LoF mutations (Figure 6). We observed alternative genetic load patterns for missense and LoF mutations in Asiatic mouflon and domesticated sheep (Figure 6). The mutation load for missense (number of homozygous and total number of alleles) was higher in Asiatic mouflon than in domestic sheep (*p* < 0.01) (Figure 6A,C,E). Still, the mutation load for LoF (number of heterozygotes and number of homozygous) was not significantly different between Asiatic mouflon and domesticated sheep (Figure 6B,D,F). We also observed lower mutation load for missense in improved breeds than in native populations (*p* < 0.01) (Figure 6C,E), and there was no significant difference in mutation load for LoF among improved breeds than native populations (*p* > 0.05) (Figure 6D,F). The results suggested that domestication may purify weak genetic load (missense mutation) more efficiently in domestic and improvement populations. 

## 4. Discussion

The domestication and improvement processes exert different evolutionary forces on sheep, such as bottleneck, artificial selection, and the relaxation of selective pressures. They may reshape genomic characteristics (e.g., genetic diversity, mutation burden) [6,17,38,39]. Then, the diffusion of domesticated sheep with humans and dramatic growth in population size may be a risk to the survival of relative species by competing resources, isolating habitat, and genetic erosion [19,40,41]. Understanding how evolutionary factors (e.g., *Ne*) cause genetic diversity and deleterious mutations is vital to sheep breeding and wild species conservation [2,42]. 

### 4.1. Genetic Diversity and Mutation Burden among Wild Species

Nucleotide diversity and heterozygosity are pivotal metrics in assessing the conservation status of wild species, exerting influences on factors such as fitness, inbreeding, and genetic load [43]. We calculated a suite of genetic parameters, including nucleotide diversity (π), heterozygosity, *F*_ROH_, *F*-index, and genetic load (Tables S3 and S4). Significant variations in genetic diversity and mutation burden were observed among species (Figure 1 and Figure 2). These observations are consistent with the population structure observed in Chen et al. and Upadhyay et al., in which Bighorn sheep, Thinhorn sheep, and Snow sheep form a cluster; Urial sheep, Asiatic mouflon, and European mouflon form a cluster; and Argali form a unique cluster [11,44]. We made an intriguing observation of subgenus *Pachyceros*, all species of which presented similar genetic diversity (lower genetic diversity and heightened inbreeding coefficients) (Figure 1) and deleterious mutation (increased genetic load) patterns. The alternative pattern among subgenus *Pachyceros* and other species within *Ovis* is consistent with the phylogeny of *Ovis*. Two genetic clades were clarified based on multiple accounts [44,45,46], and three species within subgenus *Pachyceros* dramatically declined in population size ~80–250 thousand years ago [11], which may be caused by the genomic landscape. These findings emphasize the need for augmented conservation efforts towards subgenus *Pachyceros.* All three species are still categorized as “Least Concern” by the IUCN Red List (https://www.iucnredlist.org/, last accessed on 20 July 2023).

European mouflon presents a distinctive case, with the lowest genetic diversity, the highest inbreeding coefficient, and a lower genetic load (Figure 1 and Figure 2). The results could be ascribed to feral species’ specific history [13,47,48]. The feralization of European mouflon is accompanied by founder effects and bottleneck events, reducing genetic diversity and increasing inbreeding and genetic load. However, we did not observe elevated genetic load in European mouflon, which could have been subjected to a combination of genetic drift and the purging of deleterious mutations through increased inbreeding and purifying selection in small populations [4]. Argali exhibits intermediary levels of genetic diversity and genetic load compared to the other species (Figure 1 and Figure 2). This suggests the potential role of Argali as a genetic bridge facilitating gene flow between subgenus *Pachyceros* and other species within *Ovis* [11,44].

### 4.2. N_e_ and Genetic Load

The effective population size (*N*_e_) is a critical parameter in population genetics and is indirectly related to genetic load via genetic drift [49]. However, patterns of inconsistency were observed. Smaller *N*_e_ might purge deleterious mutations via purifying selection and genetic drift in Island foxes and kākāpō [4,5] and enhance the accumulation of mutation burden in killer whale and Island songbirds [1,50]. Conversely, some studies suggest that larger populations could harbor a higher potential genetic load, while smaller populations might have a higher realized genetic load [35].

Our analysis demonstrated that recent effective population size has no relationship with genetic load across seven species within *Ovis* (Figure 4). The three species in subgenus *Pachyceros* tend to carry a higher genetic load and lower genetic diversity patterns (Figure 1 and Figure 2). In addition, we identified a specific species—European mouflon—with the lowest *Ne*, lower genetic diversity, and higher inbreeding but lower genetic load (missense and LoF). This suggests that the small *N*_e_ of European mouflon reduced the number of deleterious mutations more efficiently compared to other species within the genus *Ovis* [13]. We also found extensive variants of *N*_e_ among Bighorn sheep, Thinhorn sheep, and Snow sheep (Figure 4), and an almost identical demographic history ~80–250 thousand years ago [11,44]. The result, combined with similar genetic diversity and genetic load patterns in subgenus Pachyceros, suggested that the dramatic decline in population sizes throughout history shaped *Ovis*’s genetic diversity and genetic load pattern. 

### 4.3. Cost of Domestication

In the “cost of domestication” hypothesis, domestication and the improvement processes are expected to reduce genetic diversity and increase genetic load [51]. Our results revealed that the domestication process in sheep led to a decrease in genetic diversity and an increase in inbreeding (Figure 5). Conversely, the improvement process resulted in lower π and higher heterozygosity and reduced inbreeding in improved populations compared to native breeds. These observations could be attributed to hybridization in the improved process. For example, Merino sheep originated from hybridization involving multiple breeds [52], which may explain the increased heterozygosity and decreased inbreeding. Similar patterns have been observed in other livestock species, such as pigs [53] and cows [54], suggesting that hybridization can enhance the heterozygosity level of breeds.

Our analysis revealed that domestication and improvement did not influence large-effect mutations (LoF mutations) (Figure 6B,D,F), which could be attributed to a strong purge of large-effect mutations in all populations [53]. However, we observed a reduction in weak genetic load in native and improved populations (Figure 6C,E); the pattern coincides with smaller populations purging weak genetic load (missense) more efficiently [54]. Further validations, such as simulations, are necessary in order to explore this relationship comprehensively.

## 5. Conclusions

This study illuminates new insights into the impact of effective population size, domestication, and improvement processes on genetic diversity and mutation burden. Our research demonstrates that genus *Ovis*’s genetic load and diversity have no discernible impact on recent *N*_e_. We also observed lower genetic diversity in domesticated sheep than in Asiatic mouflon and counterintuitive patterns of genetic load, i.e., lower missense mutations in domestic sheep than in Asiatic mouflon, indicating that domestication and improvement processes lower the weak genetic load (missense) more efficiently. Collectively, this study underscores the need for a more nuanced and comprehensive understanding of these multifaceted relationships and emphasizes the critical role of genomics in species conservation and animal breeding.

## Figures and Tables

**Table 1:** Summary of sample information.

Breed/Species	Abbr.	Group	Number of Individual	Country	Data
Europe Mouflon (*Ovis musimon*)	EMUF	Wild species	3	Finland	WGS
Asiatic Mouflon (*Ovis orientalis*)	AMUF	Wild species	31	Iran	WGS
Urial (*Ovis vignei*)	URIS	Wild species	9	Iran	WGS
Argali (*Ovis ammon*)	ARGS	Wild species	8	China	WGS
Snow (*Ovis nivicola*)	SOWS	Wild species	8	Russia	WGS
Thinhorn (*Ovis dalli*)	THNS	Wild species	6	Canada	WGS
Bighorn (*Ovis canadensis*)	BIGS	Wild species	6	Canada	WGS
Tibetan	TBT	Native breed	15	China	WGS
Finn	FIN	Native breed	10	Finland	WGS
Shetland	SHE	Native breed	7	The Netherlands	WGS
Chinese Merino (super fine wool)	MSF	Improved breed	10	China	WGS
Chinese Merino (fine wool)	MFW	Improved breed	10	China	WGS

## Data Availability

All data were downloaded from NCBI.

## Supplementary Materials

The following supporting information can be downloaded at: https://www.mdpi.com/article/10.3390/genes14101977/s1, Table S1: Sample informations in this study; Table S2: Effective population size (*Ne*) of 7 wild *Ovis* species before 100 generations; Table S3: Summary of genomic diversity of 7 wild *Ovis* species; Table S4: Wilcoxon test of AMUF, Landraces and Improved populations for genetic diversity and mutation load.

## Code Availability:

All scripts used for this work were performed using open-source software tools and are available from the corresponding authors upon request.
